# Evaluating the Efficacy of 30 Different Essential Oils against *Varroa destructor* and Honey Bee Workers (*Apis mellifera*)

**DOI:** 10.3390/insects12111045

**Published:** 2021-11-21

**Authors:** Marian Hýbl, Andrea Bohatá, Iva Rádsetoulalová, Marek Kopecký, Irena Hoštičková, Alena Vaníčková, Petr Mráz

**Affiliations:** 1 Faculty of Agriculture, University of South Bohemia in Ceske Budejovice, Studentska 1668, 370 05 Ceske Budejovice, Czech Republic; mario.eko@seznam.cz (M.H.); bohata@zf.jcu.cz (A.B.); mkopecky@zf.jcu.cz (M.K.); jelini00@zf.jcu.cz (I.H.); 2 Faculty of AgriSciences, Mendel University in Brno, Zemedelska 1, 613 00 Brno, Czech Republic; radsetoulalova.iva@gmail.com; 3 Aromaterapeutická KH a.s., Kšice 11, 349 01 Stříbro, Czech Republic; vanickova@karelhadek.eu

**Keywords:** acaricidal effect, complete exposure bioassay, honey bee, screening, Varroa mite

## Abstract

**Simple Summary:**

Worldwide, mass losses of honey bee colonies are being observed more frequently due to Varroa mite infestation. Therefore, varroosis is considered a major problem in beekeeping participating to a large extent in colony collapse disorder. Except for direct damage of bees and suppressing their immune system caused by parasitism, Varroa mites transfer viral particles straight to bee hemolymph which can have a fatal impact. To control the mite population, several acaricidal treatments are used. Commonly used treatments are synthetic acaricides with a high risk of developing Varroa resistance population and contamination of bee products by acaricidal residues. Other commonly used treatments are organic acids, which are increasingly associated with damage of brood, adult bees, and premature deaths of queens. Therefore, in this study, we evaluated the varroacidal effect of 30 individual essential oils. The toxicity of the most effective oils selected by screening was subsequently tested on Varroa mites and adult honey bee workers simultaneously. In addition, the main components of these essential oils were specified. Several essential oils were proven to be effective against the adult female of Varroa mites and at the same dose safe for adult honey bee workers under laboratory conditions, especially manuka, peppermint, oregano, litsea, and cinnamon.

**Abstract:**

Essential oils and their components are generally known for their acaricidal effects and are used as an alternative to control the population of the *Varroa destructor* instead of synthetic acaricides. However, for many essential oils, the exact acaricidal effect against Varroa mites, as well as the effect against honey bees, is not known. In this study, 30 different essential oils were screened by using a glass-vial residual bioassay. Essential oils showing varroacidal efficacy > 70% were tested by the complete exposure assay. A total of five bees and five mites were placed in the Petri dishes in five replications for each concentration of essential oil. Mite and bee mortality rates were assessed after 4, 24, 48, and 72 h. The LC_50_ values and selectivity ratio (SR) were calculated. For essential oils with the best selectivity ratio, their main components were detected and quantified by GC-MS/MS. The results suggest that the most suitable oils are peppermint and manuka (SR > 9), followed by oregano, litsea (SR > 5), carrot, and cinnamon (SR > 4). Additionally, these oils showed a trend of the increased value of selective ratio over time. All these oils seem to be better than thymol (SR < 3.2), which is commonly used in beekeeping practice. However, the possible use of these essential oils has yet to be verified in beekeeping practice.

## 1. Introduction

The main threat for beekeeping is a varroosis caused by the obscure ectoparasitic mite *Varroa destructor* Anderson and Trueman (Acari: Varroidae) [1,2]. The mite feeds on the fat body of bees [3] and thus reduces the weight and fitness of newly emerging adult bees, affects cuticle properties [4] and suppresses the immune response system [5]. In addition, *V. destructor* acts as a vector of viruses [6], including deformed wing virus, Kashmir bee virus, and Israeli acute paralysis virus [7,8,9,10,11]. These viruses are transmitted in large doses directly to the hemolymph of the bee brood and adult honey bees [5]. Infected individuals weaken, their lifespan is shorter, and the infection can lead through visible damaged bodies and wings [12,13] to the colony collapse at the final stage [1,6]. For these reasons, and also due to its almost worldwide distribution [1], *V. destructor* is associated with colony collapse disorder (CCD) [14,15].

Reproduction of the *V. destructor* mite is closely related and synchronized with the development of the bee brood [16]. Adult mated female mites enter the bee colony attached to worker and drone bees, usually hidden under the sternites of bees, and then enter brood cells only several hours before capping. Varroa mites can find the adult honey bee workers and bee brood before capping based on chemical communication [1]. In colonies highly infested (>7%) with *V. destructor* [1], the bee population is significantly reduced, and eventually, the entire colony crashes unless the mite population is treated [17]. Colonies in temperate areas must therefore be treated several times in a year against *V. destructor* to keep mite populations at acceptable levels [18].

For a long time, the use of synthetic chemicals has been considered the most effective way to control *V. destructor* [19], especially pyrethroids and organophosphates [20]. Except for their declining efficiency due to emerging resistance against *V. destructor* [21,22,23], excessive use of these compounds has, in many cases, also led to contamination of bee products [24,25,26], especially honey and beeswax [24]. This could endanger the health of bees and humans with potential sublethal doses of pesticide residue mixtures [27,28]. As a result, the idea of finding new and safer ways to control the parasite is spreading. Thus, natural products offer a very desirable alternative to synthetic products. Interest in these substances is still growing because they are generally cheap and have lower health risks for humans and bees [29].

In response, beekeepers are showing a growing interest in treatments that work on physical intolerance rather than enzyme degradation, as is the case with synthetic acaricides, to which resistance develops. Therefore, natural chemicals such as organic acids, essential oils, and their derivatives are increasingly used [30,31]. However, several studies suggest that the use of organic acids against Varroa may be harmful to bees. For example, damage and removal of open and capped brood are most commonly observed [32,33]. In addition, permanent damage to the digestive and excretory organs and glands of bees was described [34,35], as well as damage to the queen or often even premature death [36,37], or a decrease in the pH of honey during the following season [38].

Another possible way to reduce Varroa mites is essential oils (EO) [39]. According to The Commission of the European Pharmacopoeia, EOs are odorous products, usually with a complex composition, obtained from a botanically defined plant raw material by steam distillation, dry distillation, or a suitable mechanical process without heating. They are usually separated from the aqueous phase by a physical process that does not significantly affect their composition. EOs are lipophilic and may contain over 100 different plant secondary metabolites (terpenoids and phenylpropanoids, monoterpenes, sesquiterpenes, aldehydes, alcohols, etc.) [40]. Among natural substances, essential oils represent one of the most promising alternatives to synthetic chemicals [41,42,43,44,45,46,47,48], with minimal side effects [49,50,51,52]. The effectiveness of EOs against *V. destructor* is comparable to organic acids, but the application of EOs causes a lesser degree of stress in bees than the application of organic acids [29].

In addition to acaricidal effects, the application of EOs into hives often also causes antimicrobial effects, which can lead to an overall improvement in the health status of honey bee colonies [53]. Most research suggests that essential oils may be a useful alternative to maintaining a low level of mite infestation in hives [39,54,55,56,57]. Adamczyk et al. [58] concluded that the presence of residues of essential oil components in honey samples does not pose a hygiene risk or a risk to human health.

Despite the promising acaricidal effects of various EOs found in vitro [54,55,57], only a fraction of them has been tested under beehive conditions [39]. This could be the reason why EOs have not yet been included in many commercial formulations, with the exception of some cases [53].

The aim of the study was therefore to determine the acaricidal effect of a large number of selected EOs against Varroa mites, as well as their effect on honey bees in vitro, which select the most promising essential oils for the in vivo experiments. In addition, the most promising EOs were quantified for their major components.

## 2. Materials and Methods

### 2.1. Biological Material and Essential Oils

*V. destructor* mites and honey bees (*Apis mellifera*) used in this study were obtained from the experimental apiary of the Faculty of Agriculture, the University of South Bohemia in České Budějovice, (Czech Republic). To rear mites, 4 honey bee colonies were infested by Varroa mites and untreated for over 12 months. From the infested beehives, the bees were collected in a mesh container by sweeping from the brood frames and subsequently exposed to CO_2_. After anesthesia of the bees, the vessel was closed and shaken until mites fell over the mesh bottom [59]. Thus, a sufficient number of adult vital female mites were collected. Mites showing signs of defect, newly molded, or poorly mobile were eliminated.

A total of 30 essential oils (EO) were obtained from company 1. Aromaterapeutická KH a.s. (Czech Republic). The list of EOs, their abbreviations, Latin names, and part of used plants are given in Table 1.

### 2.2. Screening of Essential Oils for Their Acaricidal Activity

To evaluate EO acute toxicity on *V. destructor*, a glass-vial residual bioassay was used [60]. Each tested product was diluted in acetone (0.375 µL EO/500 µL acetone). This solution was pipetted into a 10 mL glass vial. Glass vials were rolled on their side until the acetone evaporated and EOs created a cohesive film. Then, 5 vital female adult mites were placed in each glass vial using a fine brush. The glass vials were sealed and placed in a dark room at 25 °C and 65% RH. For each treatment, including acetone as a negative control and thymol (THM) as a positive control; 5 repetitions were provided (each repetition in an individual glass vial).

The mortality rates of Varroa mites were evaluated 2 and 4 h after the treatment, and the efficacy of tested EOs was determined [55]. The mites were transferred to a white pad and encouraged to move with the brush. Mites that did not move even after repeated brushing were considered dead.

### 2.3. Complete Exposure Bioassay

EOs showing >70% mite mortality in the screening test were subjected to further testing in the complete exposure method [61]. Dosages of EOs were prepared based on the mortality of previous experiments with honey bees (data not included). A selected amount of EOs was diluted in 0.5 mL of acetone. This solution was pipetted on the bottom of the Petri dish and subsequently covered with filter paper (Whatman 1). After evaporation of the solvent, five vital adult honey bee workers were placed in each Petri dish, together with five vital female adult Varroa mites. Positive control (thymol) and negative control (acetone only) were included. Altogether, 5 replicates were established for each treatment (each repetition in an individual Petri dish). Immediately after the establishment, the Petri dishes were transferred to an incubator (28 °C ± 0.5). Honey bee and mite mortality were assessed after 4, 24, 48, and 72 h. The values of LC_50_ and selectivity ratio (SR) were calculated. SR is a ratio between mite and bee toxicity, and it was determined according to the following formula: SR = LC_50 A. mellifera_/LC_50 V. destructor_.

### 2.4. Assessment of the Main Components of the Examined EOs

Samples of essential oils were analyzed diluted 1:10,000 in hexane by GC MS/MS system consisting of TriPlus autosampler, Trace GC Ultra gas chromatograph equipped with a TG-5MS fused silica capillary column, 30 m × 0.25 mm × 0.25 μm and coupled to a mass spectrometer TSQ Quantum XLS all from Thermo Fischer Scientific, Cleveland, OH, USA. Helium was used as a carrier gas at 1.0 mL/min. A total of 1 μL of the sample was injected into the SSL injector in the splitless mode set at 280 °C. The oven temperature was programmed as follows: start at 40 °C and held for 5 min, then increased to 150 °C at a rate of 3 °C/min and held for 0.5 min, then increased to 250 °C at a rate of 10 °C/min, then increased to 290 °C at a rate of 25 °C, and finally maintained at 290 °C for 10 min. The temperature of the transfer line was held at 250 °C, and the ion source was operating at 200 °C. TIC mode was performed on Q1 at 70 eV of ionization energy and mass range 50–450 m/z. To exclude congestion of detector the scanning was performed after 6 min of injection. The data were processed in Thermo Xcalibur 3.0.63 (Thermo Fisher, Waltham, MA, USA). Component identification was made based on comparison with the NIST Mass Spectral Search Program library v 2.0 f (Thermo Fisher). The quantification was achieved based on Q3 SIM mode focused on fragmentation ions of desired compounds and also via an external calibration curve. The Thujone (Sigma Aldrich, St. Louis, MO, USA) was used as an internal and also external standard.

### 2.5. Statistical Analyses

Statistical analyses of the screening of essential oils, including graphical outputs, were processed in STATISTICA (version 14, TIBCO Software Inc., Palo Alto, CA, USA, 2021), specifically, the analysis of variance procedure ANOVA, preceded by a normality test. Statistical significance was tested at a level of significance = 0.05.

Probit analyses were calculated in XLSTAT (Addinsoft, 2016) incorporating natural mortality into the analyses. The concentration of essential oils was transformed logarithmically. LD_50_ with 95% confidence intervals (*p* < 0.05) were fitted.

The in vitro effect of each active substance on mortality of both Varroa mite and honey bees was analyzed by the test of hypothesis for two samples representing independent binomial experiments, and the acaricidal effects of active substances were subsequently evaluated (GenStat 17). Significant differences among substances were stated where *p* ≤ 0.05.

## 3. Results

### 3.1. Screening of Essential Oils for Their Acaricidal Activity

**Figure 1:**
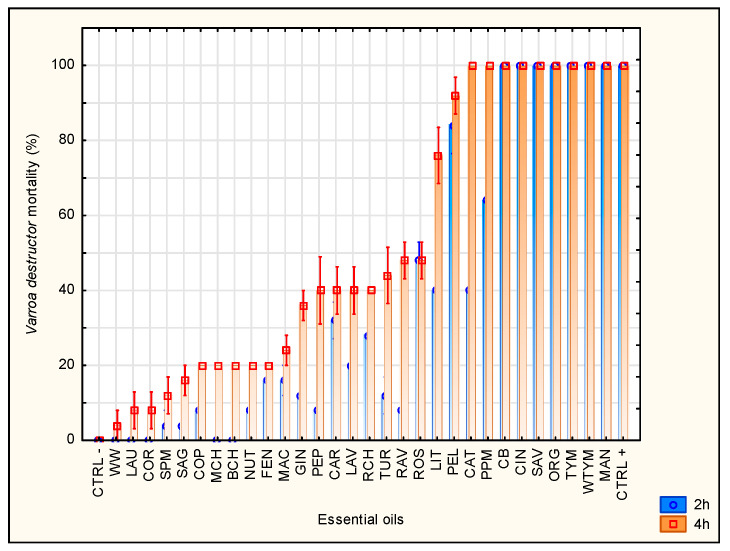
Mortality rates of *Varroa destructor* in glass vial bioassay after 2 and 4 h of EO exposition. The error bars denote standard deviation. Full name of each abbreviation is shown in Table 1.

All 30 EOs were screened for acaricidal effect in glass vials (Figure 1). Based on these results, the EOs were divided into three categories according to their efficacy. A total of 11 EOs showed a high acaricidal efficacy (>70%) and were further tested on Petri dishes (complete exposure assay) simultaneously with honey bees and mites. These were MAN, TYM, WTYM, ORG, SAV, CIN, CB, PPM, CAT, PEL, LIT, and THM as a positive control. The category of moderately effective oils (30–50%) includes ROS, RAV, TUR, RCH, LAV, CAR, PEP, and GIN. The last category with an efficiency of less than 30% includes NUT, FEN, MAC, BCH, MCH, COP, SAG, SPM, COR, LAU, and WW. Oils showing less than 70% efficacy were further tested.

### 3.2. Complete Exposure Bioassay

The complete exposure bioassay reveals that EO from MAN showed by far the lowest LC_50_ value against Varroa mites both after 4 and 72 h of exposure. EOs from TYM, ORG, and control THM also had a low LC_50_ value after 72 h of exposure. A moderate LC_50_ value after 72 h showed PPM, SAV, WTYM, CB, and CIN. EO from PEL and CAT showed a relatively high value. The lowest LC_50_ value for bees after 72 h of exposure had EOs from MAN, TYM, and control THM, slightly high values had CB and ORG. In contrast, bees were most tolerant of EOs from CAT, PPM, LIT, and PEL (Table 2).

The selectivity ratio was calculated based on the LC_50_ values. The estimated LC_50_ values, including standard deviation obtained at each observation time and selectivity ratio for every treatment, are shown in Table 2. By far, the highest value of the selective ratio was reached after 72 h of exposure to EO from PPM (SR = 9.65) and MAN (SR = 9.33). ORG (SR = 5.83) and LIT (SR = 5.35) also reached high values at 72 h. All these four oils had an increasing SR value over time. In contrast, TYM and PEL oils had the highest SR value after 4 h of exposure (SR = 6.85; SR = 6.12). A significant decrease in this value was observed in the following measurements. Moderately high SR values were observed after 72 h of exposure in CAT, SAV, and WTYM, which showed an increasing tendency of SR value in time (SR = 4.55; SR = 3.39; SR = 3.36). A moderate-to-high SR value was also observed in THM (positive control). After 4 h of experiment, THM showed even one of the highest SR values (SR = 4.11), however, with a declining trend of SR values in time. A constantly low value of SR was observed with CB, as in each measurement SR was less than 3. Similarly, CIN also had a low value of SR, and with the exception of the last measurement after 72 h of exposure, the level of SR increased significantly (SR = 4.54).

The main components and their quantity of the most effective EOs were assessed (Table 3). The most frequent substances were carvacrol and p-cymene.

## 4. Discussion

Investigation of the acaricidal activity of essential oils is a major concern of many scientific studies. However, large-scale screening of a number of EOs is rare, and most of the effort is devoted to an individual or a small number of selected oils, such as thyme, clove bud, or oregano [55,56,62]. In this study, the acaricidal effect of 30 EOs on *V. destructor* mites was assessed by the glass vials bioassay (Figure 1), which represents a simple and quick way to determine the effectiveness of individual EOs [60].

Thymol, as a derivate of thyme, was included in the screening as a positive control, as it is commonly used in beekeeping practice as an acaricide [63]. However, thymol could have some negative effects on bees, including toxicity on bee brood, metabolic disorders, changes in bee’s behaviors, etc. [64,65,66,67,68,69,70,71].

In the experiment, after 4 h of exposure, all EOs showed either the same or higher acaricidal effect than after 2 h. Based on the results of mortality after 4 h of exposure, the individual EOs were divided into three categories according to their effectiveness: highly effective, moderately effective, and minimally effective. The oils in the highly effective group, including MAN, WTYM, TYM, ORG, SAV, CIN, CB, PPM, CAR, PEL, and LIT, were further tested. Almost all oils in this group were able to kill 100% of mites after 2 h, with the exception of PPM, CAR, PEL, and LIT. The EOs from the moderately effective group have still the potential to participate in the mite control; however, a higher dose or applying a certain mixture showing a stronger synergistic effect would be needed. From the group of moderately effective EOs, the best acaricidal activity belonged to ROS, RAV, and TUR. The oils from the minimally effective group showed a very low varroacidal effect, and therefore, they were not suitable for further testing. Especially WW, LAU, and COR appear to be ineffective.

The 11 EOs from the highly effective group were further tested in order to determine the most suitable EOs for the best potential use in beekeeping practice. In addition to mite toxicity, the bee tolerance was necessary to be evaluated. Therefore, the method of complete exposure assay [61] was chosen, which allows the evaluation of selectivity ratio (SR), the most telling data for this purpose, in addition to LC for mites and bees [57].

In the complete exposure bioassay, after 4 h of exposure, only MAN and TYM showed a higher level of mite toxicity than THM (control). After 72 h of exposure at the end of the experiment, MAN, TYM, and ORG showed higher mite toxicity. The higher degree of toxicity of the above-mentioned EOs, compared with THM, is probably due to the content of other active substances (carvacrol, p-cymene, calamenene, leptospermone), which can additionally act synergistically [56,72]. While the varroacidal effect has already been described for TYM and ORG [55,56,62], for MAN, it has not been described yet. However, its antimicrobial and also acaricidal effects against other mite species (*Dermatophagoides* and *Tyrophagus*) have been observed [73,74]. Regarding bee toxicity, only EOs from CB and MAN were more toxic than THM after 4 h of exposure. After 72 h, at the end of the experiment, a higher degree of toxicity was observed only in EO from MAN. Thus, the results indicate higher toxicity of THM to Varroa mites but also to honey bees [55,57].

The ratio between mite and bee toxicity is defined as selectivity ratio (SR) values. At the beginning of the experiment, after 4 h of exposure, THM showed an SR value of 4.107, which was better than most EOs tested. Higher SR value was observed only at PEL (6.120) and TYM (6.848). However, with the duration of exposure, the SR value of THM decreased. After 72 h of exposure, the value was only 3.198. This can be explained by a decrease in mite toxicity, an increase in bee toxicity, or a combination of both in time. [64,75]. A similar trend was observed for PEL and TYM. In both EOs, the SR value also decreased with the duration of exposure; however, in both EOs, the SR value was always higher, compared with THM. This declining trend in the SR value with increasing exposure time for TYM is consistent with the results of Damiani et al. [62] and is probably due to the high thymol content that is characteristic of thyme [76]. This declining trend in the SR value indicates the potential unsuitability of EOs with these properties, and these EOs need to be subjected to further testing.

Stable to slightly fluctuating development of SR values depending on the duration of exposure was observed at SAV and CB. The initial values at the beginning of the measurement were very similar to the values at the end of the experiment and do not change significantly during the experiment. However, the SR value was significantly lower in CB than in THM, which is in accordance with the results of Damiani et al. [62], and in the case of SAV, the SR values are similar to THM. In the other tested EOs, an opposite trend was observed, and the SR increased with the time of exposure.

The best SR value after 72 h was determined at EOs from PPM (SR = 9.651) and MAN (SR = 9.333), followed by ORG (SR = 5.830) and LIT (SR = 5.354). From the results of Nazer and Al-Abbadi [77], it seems EO from PPM is more suitable to control varroosis than THM in vivo. The same conclusion can be drawn from the results of Damiani et al. [62] in the case of ORG in in vitro conditions. There is still a lack of varroacidal data from MAN and LIT in the literature; however, a strong antimicrobial effect against *Clostridium*, *Bifidobacterium*, *Escherichia*, *Staphylococcus*, *Lactobacillus*, and an acaricidal effect against *Dermatophagoides* and *Tyrophagus* is known for both EOs [73,74,78].

A very good result after 72 h was also observed at EOs from CIN (SR = 4.542) and CAT (SR = 4.552). CIN is proposed as a suitable option for reducing the population of *V. destructor*. In addition, CIN has a strong repellent effect on *V. destructor* mites and is also gentle on bees [39]. The suitability of CAT for further testing in the beehive conditions is also proved by its strong inhibitory effect against *Ascosphera apis* and *Paenibacillus larvae* [79,80]. A slightly lower SR value, but still higher than THM, was observed in EO from WTYM (SR = 3.358).

Since the chemical composition of EOs is influenced by many factors (geographical origin, part of the plant, agrotechnics, genotype, extraction technology, etc.), it is necessary to know their composition to interpret the effect of individual EOs [78].

According to SR of EOs from PPM and MAN, they seem to be the most promising oils against *V. destructor*. The most represented substances in PPM were limonene, menthol, and α-pinene. Limonene has been shown to be effective in reducing the population of *V. destructor* at a colony level [81] and has strong antimicrobial effects [82]. Varoacidal [55] and antimicrobial effects have also been reported for menthol [83], whereas α-pinene is known for its inhibitory effects on bacteria [84]. In addition, it can also be produced in larger quantities by genetically modified bacteria [85]. In the case of MAN, calamenene and leptospermone were the most abundant constituents. Celemonene-containing oils show high antimicrobial and fungicidal activity and are effective against a wide range of pathogens, including methicillin-resistant *Staphylococcus aureus* (MRSA) strains, and also have high antioxidant activity [86]. Leptospermone is known for its bactericidal, antiviral, and acaricidal effects [73,74].

Other EOs with suitable results were LIT and ORG. The main components of LIT were citral (A and B) and limonene. The findings of Liu et al. [87] agree with ours that citral is the main component of litsea and has a strong aroma and strong antimicrobial effects [88] against both, gram-positive and gram-negativebacteria [78]. At ORG, carvacrol was absolutely dominant and is known for its significant acaricidal and antimicrobial effects. In addition, it also has anti-inflammatory and antimutagenic, and antigenotoxic effects [89]. CIN and CAT also showed a significant acaricidal effect. The main component of CIN was cinnamaldehyde, to which Conti et al. [39] attributed the main varroacidal effects in cinnamon EO. It also has antibacterial effects [90]. Ceratol and α-pinen were predominant in CAT. The last EO with better results than THM was WTYM, with an almost balanced representation of thymol, carvacrol, and p-cymene.

## 5. Conclusions

The results based on selectivity ratio (SR) value for individual EOs showed that potential best EOs for Varroa control are PPM and MAN, followed by ORG and LIT. Other suitable candidates seem to be CAT, SAV, WTYM, and CIN. All these oils showed better SR values at the end of the experiment than THM (control group), which is used in beekeeping practice. Additionally, these oils showed a trend of an increased value of the selective ratio.

Thymol showed very good SR at the beginning of the experiment, but this value declined with all following measurements. At the end of the experiment, the SR value was lower than the values of most tested essential oils. This trend was also observed in EOs from PEL and TYM.

Except for well-known substances such as thymol, menthol, and carvacrol, other components appear to be potentially interesting for the control of Varroa, especially citral, limonene, calamenene, leptospermone, p-cymene, and cinnamaldehyde, as the main compounds of the most effective EOs.

## Figures and Tables

**Table 1:** The list of essential oils, abbreviations, Latin names, and part of the used plants.

English Name	Abbreviation	Latin Name	Part of Plant
Black pepper	PEP	*Piper nigrum*	berry
Blue chamomile	BCH	*Matricaria chamomilla*	flower
Carrot	CAT	*Daucus carota*	seeds
Cinnamon	CIN	*Cinnamomum zeylanicum*	bark
Clove Bud	CB	*Eugenia caryophyllata*	leaves, buds, and twigs
Copaiba	COP	*Copaifera reticulata*	resin
Coriander	COR	*Coriandrum sativum*	seeds
Fennel	FEN	*Foeniculum vulgare*	seeds
Ginger	GIN	*Zingiber officinale*	rhizome
Green cardamom	CAR	*Elettaria cardamomum*	seeds
Laurel	LAU	*Laurus nobilis*	leaves
Lavender	LAV	*Lavandula angustifolia*	flowering herb
Litsea	LIT	*Litsea cubeba*	fruits
Mace	MAC	*Myristica fragrans*	flower
Manuka	MAN	*Leptospermum scoparium*	leaves and twigs
Maroc chamomile	MCH	*Ormenis multicaulis*	herb
Nutmeg	NUT	*Myristica fragrans*	seeds
Oregano	ORG	*Origanum vulgare*	herb
Pelargonium	PEL	*Pelargonium graveolens*	leaves and flowers
Peppermint	PPM	*Mentha piperita*	herb
Ravensara	RAV	*Ravensara aromatica*	leaves and twigs
Roman chamomile	RCH	*Anthemis nobilis*	flower
Rosemary	ROS	*Rosmarinus officinalis*	herb
Sage	SAG	*Salvia officinalis*	leaves
Savory	SAV	*Satureja montana*	herb
Spearmint	SPM	*Mentha spicata crispa*	flowering herb
Thyme	TYM	*Thymus vulgaris*	herb
Turmeric	TUR	*Curcuma longa*	root
Wild thyme	WTYM	*Thymus serpyllum*	herb
Wormwood	WW	*Artemisa absinthium*	herb

**Table 2:** Complete exposure bioassay. LC_50_ (µL) of essential oils on *V. destructor* and *A. mellifera* and their selectivity ratio in a monitored period. Green highlighting means low value of selectivity ratio (<3), yellow highlighting means moderate value of selectivity ratio (3–5), and red highlighting means high value of selectivity ratio (>5).

EO	Species		4 h			24 h			48 h			72 h	
		LC_50_	95% CL		LC_50_	95% CL		LC_50_	95% CL		LC_50_	95% CL	
THM	*V. destructor*	1.505	1.180	1.937	0.834	0.629	1.052	0.660	0.475	0.846	0.660	0.475	0.846
	*A. mellifera*	6.181	5.074	7.847	4.090	3.189	6.759	2.427	2.097	2.871	2.112	1.940	2.320
	Selectivity ratio	4.107			4.903			3.675			3.198		
CAT	*V. destructor*	10.449	6.806	34.882	4.167	2.457	6.630	3.276	1.930	4.590	2.539	1.187	3.653
	*A. mellifera*	18.607	13.845	64.136	13.048	9.588	27.137	11.557	8.855	19.447	11.557	8.855	19.447
	Selectivity ratio	1.781			3.131			3.527			4.552		
PPM	*V. destructor*	8.121	6.159	13.576	2.512	1.430	3.578	1.732	0.499	2.806	1.066	0.011	2.197
	*A. mellifera*	12.951	11.259	14.994	10.759	9.483	12.156	10.285	9.109	11.568	10.285	9.109	11.568
	Selectivity ratio	1.595			4.283			5.939			9.651		
SAV	*V. destructor*	3.825	3.165	4.918	2.008	1.323	2.754	1.459	0.626	2.075	1.364	0.417	1.996
	*A. mellifera*	11.657	9.247	16.335	5.786	4.607	7.218	5.275	4.273	6.467	4.621	3.884	5.897
	Selectivity ratio	3.048			2.881			3.615			3.386		
WTYM	*V. destructor*	8.185	5.218	22.390	2.549	1.495	8.207	2.013	0.926	7.327	1.861	0.825	5.487
	*A. mellifera*	9.074	8.106	10.780	7.517	6.606	8.265	6.512	5.958	7.494	6.250	5.603	6.897
	Selectivity ratio	1.109			2.949			3.236			3.358		
ORG	*V. destructor*	3.517	2.339	7.322	0.879	0.638	1.302	0.577	0.280	0.924	0.577	0.280	0.924
	*A. mellifera*	6.982	6.136	7.889	3.362	2.997	3.803	3.362	2.997	3.803	3.362	2.997	3.803
	Selectivity ratio	1.985			3.827			5.830			5.830		
PEL	*V. destructor*	2.798	0.113	4.758	2.291	0.247	3.825	2.402	0.804	3.532	2.272	0.788	3.311
	*A. mellifera*	17.122	13.427	27.935	12.401	10.159	17.209	9.479	8.132	11.201	9.479	8.132	11.201
	Selectivity ratio	6.120			5.413			3.945			4.171		
MAN	*V. destructor*	1.262	0.848	3.192	1.029	0.558	2.880	0.265	0.020	0.540	0.158	0.011	0.497
	*A. mellifera*	1.975	1.662	2.681	1.415	1.218	1.666	1.472	1.277	1.720	1.472	1.277	1.720
	Selectivity ratio	1.565			1.375			5.551			9.333		
LIT	*V. destructor*	4.801	3.522	7.436	2.716	1.322	4.311	2.116	0.243	3.761	1.807	0.243	2.989
	*A. mellifera*	11.660	9.524	15.222	11.590	8.994	20.096	9.207	7.721	12.115	9.678	7.255	18.278
	Selectivity ratio	2.429			4.267			4.352			5.354		
TYM	*V. destructor*	1.279	0.985	1.613	0.678	0.314	0.940	0.678	0.314	0.940	0.587	0.202	0.851
	*A. mellifera*	8.759	6.684	14.553	3.887	3.295	4.837	3.113	2.696	3.763	2.677	2.418	2.982
	Selectivity ratio	6.848			5.731			4.590			4.557		
CB	*V. destructor*	2.337	1.829	2.962	1.690	1.237	2.143	1.490	1.207	1.776	1.490	1.207	1.776
	*A. mellifera*	5.965	4.620	10.868	4.860	4.023	6.546	3.305	2.790	4.179	3.305	2.790	4.179
	Selectivity ratio	2.553			2.875			2.218			2.218		
CIN	*V. destructor*	4.321	3.163	5.979	2.820	1.577	4.002	2.529	1.370	3.590	1.543	0.829	2.484
	*A. mellifera*	10.635	9.559	11.972	7.488	6.680	8.408	7.488	6.680	8.408	7.007	5.835	8.664
	Selectivity ratio	2.461			2.655			2.960			4.542		

**Table 3:** Composition of the most effective essential oils and their constituents’ quantity (>5%).

EO	Main Components and Their Quantity (%)				
Carrot	Ceratol 30.28	α-Pinen 15.462	Sabinen 10.22	β- Caryophyllen 8.31	β-bisabolen 5.63
Peppermint	Limonen 38.02	Menthol 16.41	α-Pinen 15.92	β-Pinen 11.46	Menthon 5.65
Savory	Carvacrol 41.67	ɣ-Terpinen 35.82	p-Cymen 11.73	-	-
Wild thyme	Thymol 16.33	Carvacrol 15.38	p-Cymen 15.01	Geraniol 10.62	ɣ-Terpinen 10.30
Oregano	Carvacrol 73.50	p-Cymen 6.97	ɣ-Terpinen 6.02	-	-
Pelargonium	Citronellol 33.51	Geraniol 15.36	Citronellylformiat 7.81	Isomenthon 5.61	10-epi-g-Eudesmol 5.37
Manuka	Calamenene 17.92	Leptospermone 16.02	Flaveson 5.95	α-Selinene 4.62	-
Litsea	Citral A 39.03	Citral B 29.35	Limonen 13.74	-	-
Thyme	Thymol 40.96	p-Cymen 16.76	-	-	-
Clove Bud	Eugenol 86.62	β-Caryophyllen 10.21	-	-	-
Cinnamon	trans-Cinnamaldehyde 77.69	Eugenol 7.50	-	-	-

## Data Availability

The study did not report any data.
